# Morphologies, Preparations and Applications of Layered Double Hydroxide Micro-/Nanostructures

**DOI:** 10.3390/ma3125220

**Published:** 2010-12-09

**Authors:** Ye Kuang, Lina Zhao, Shuai Zhang, Fazhi Zhang, Mingdong Dong, Sailong Xu

**Affiliations:** 1 State Key Laboratory of Chemical Resource Engineering, Beijing University of Chemical Technology, 15 Beisanhuan East Road, Chaoyang District, Beijing, 100029, China; 2 Interdisciplinary Nanoscience Center (iNANO) and Department of Physics and Astronomy, Aarhus University, DK-8000 Aarhus C, Denmark

**Keywords:** layered double hydroxides, morphology, preparation, application

## Abstract

Layered double hydroxides (LDHs), also well-known as hydrotalcite-like layered clays, have been widely investigated in the fields of catalysts and catalyst support, anion exchanger, electrical and optical functional materials, flame retardants and nanoadditives. This feature article focuses on the progress in micro-/nanostructured LDHs in terms of morphology, and also on the preparations, applications, and perspectives of the LDHs with different morphologies.

## 1. Introduction 

Nanostructured materials have generated diverse scientific and technological interest in many potential areas of applications such as energy conversion, electronics, catalysis, optics, chemical sensing, and medicine. As one type of layered material, such as anionic clays (e.g., layered double hydroxides) and cationic clays (e.g., montmorillonite), layered double hydroxides (LDHs) [1,2,3,4] are a class of anionic clays with the structure based on brucite (Mg(OH)_2_)-like layers in which some of the divalent cations have been replaced by trivalent cations yielding positively charged sheets. LDHs can be represented by the general formula [M^II^*_1–x_*M^III^*_x_*(OH)_2_]*^x+^*(A*^n–^*)*_x/n_*·*y*H_2_O, where M^II^ and M^III^ cations occupy octahedral holes in a brucite-like layer and A*^n^*^−^ anion is located in the hydrated interlayer galleries. The identities of the divalent and trivalent cations (M^II^ and M^III^, respectively) and the interlayer anion (A*^n–^*), together with the value of the stoichiometric coefficient (*x*), may be varied over a wide range, giving rise to a large class of isostructural materials. Considered generally as promising materials [5,6,7,8,9] in view of their high chemical versatility associated with a tunable anionic exchange capacity, LDHs are widely used in commercial products as adsorbents, catalyst support precursors, anion exchangers, acid residue scavengers, flame retardants and polymer stabilizers. LDH powders, prepared by conventional coprecipitation, typically show preferential growth of *ab* faces (*i.e.*, perpendicular to the stacking direction) and microscale hexagonal platelet morphology [10]. Due to the highly active surface atoms, LDH powders are spontaneously ready to aggregate during storage and application, thus leading to limitations in their technical applicability. Therefore, it is of great interest and importance to generate alternative predefined structures. 

Important advances in LDH morphology have been made in the past years, ranging from LDH powders [11,12], spheres [13,14,15,16,17,18], nanosized LDH belt [19], fibrous structure [20], to LDH films on substrates [21,22,23,24,25,26,27]. In this feature article, we summarize the progress in fabrication of LDH structure materials with different morphologies from powder/sphere, one-dimensional (1-D) belt/fiber; to 2-D films. In addition, we focus on the applications of LDHs and offer some perspectives for future multifunctional LDH materials.

## 2. LDH Morphology

### 2.1. Powdery LDHs

**Figure 1:**
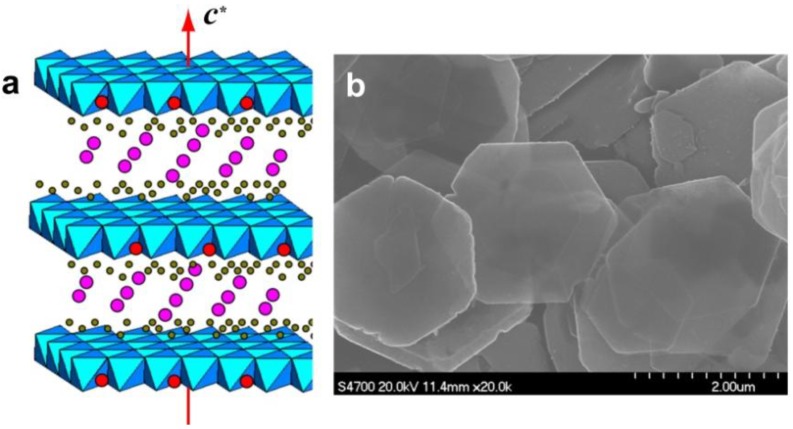
(**a**) Schematic illustration of the layered double hydroxide (LDH) structure showing the metal hydroxide octahedra stacked along the crystallographic *c* axis (indicated as a red arrow). Water (grey) and anions (pink) are present in the interlayer region. The green parts correspond to M^II^ cations and the red dots to M^III^ cations. (**b**) Scanning electron microscope (SEM) image of typical LDH crystals.

**Figure 2:**
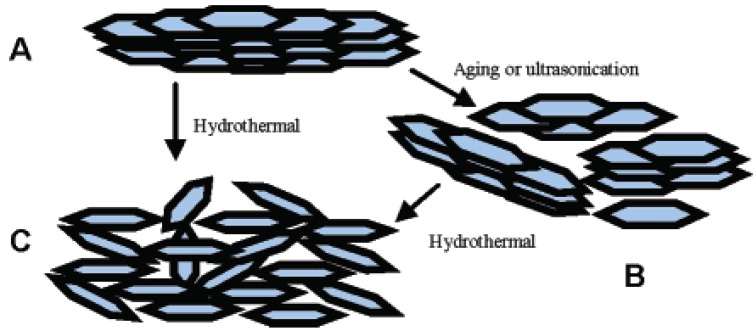
Dispersion of an LDH aggregate (**A**) into several smaller LDH aggregates (**B**) via an aging process, or (**C**) both (A and B) into individual LDH nanosheet crystallites via the new hydrothermal treatment method. Reprinted with permission from [11], © American Chemical Society, 2005.

Powdery LDHs typically show the brucite (Mg(OH)_2_)-like layered structure via a conventional co-precipitation process. When synthesized by a hydrothermal approach induced by slow hydrolysis of urea, the resulting MgAl-LDH usually exhibits hexagonal platelet morphology at the micro scale (Figure 1); this suggests the preferential growth of *ab* faces (*i.e.*, perpendicular to the stacking direction). The platelets are often observed to aggregate [10]. The microsized platelets can be fairly well prepared into nanosized crystals. Gursky *et al.* [12] obtained nanosized colloidal MgAl-LDH particles through coprecipitation from metal salts dissolved in methanol in the presence of NaOH. The approach was extended to prepare Al^3+^-based LDHs, such as CoAl-, ZnAl-, and NiAl-LDH. Xu *et al.* [11] also prepared colloidal MgAl-LDH by a fast coprecipitation followed by controlled hydrothermal treatment (Figure 2). The obtained LDH platelets had the dimensional sizes ranging from 50 to 300 nm. The approach was also extended to prepare other types of LDHs, including transition metal such as Ni^2^^+^, Fe^2^^+^, Fe^3^^+^, and Gd^3^^+^. To control the growth environment of LDH, such as by using microemulsion, O’Hare *et al.* [28,29,30,31] prepared very small LDH particles in isooctane-sodium dodecyl sulfate/aqueous solution and also obtained LDH monolayer with a thickness of 14 Å. In addition, nanosized LDH platelets can be synthesized in a large-amount production using a method involving separate nucleation and aging steps (SNAS) [32,33,34], the key features of which are a very rapid mixing and nucleation process in a modified colloid mill followed by a separate aging process. The major advantages of this method for the preparation of LDHs can be summarized as follows: (1) the synthesis process is simple to carry out, (2) small crystallite sizes can be obtained, (3) the crystallite size distribution of the product is much narrower than that obtained using conventional coprecipitation methods. With regard to types of LDHs, general LDHs are able to be prepared, such as Al^3+^-based and transmission metal cation-based LDHs. Using this method, numerous nanosized LDH building blocks were prepared, even in an organic/water solvent system for preparation of CaAl-LDH nanoplatelets without byproduct of calcium carbonate, which could be attributed to the ability of organic media (such as ethanol) to prevent carbon dioxides from entering the reaction solution [34].

### 2.2. Spherical LDHs

**Figure 3:**
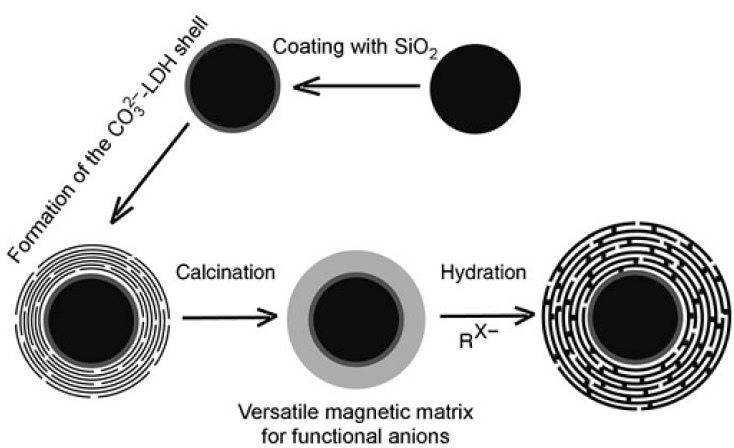
Synthesis of the magnetic Fe_3_O_4_ core/LDH shell composite structure. Reprinted with permission from [18], © Wiley-VCH, 2009.

Spherical structure is of interest for a number of different applications, such as catalysts, sorbents, lithium-ion battery electrodes, and carriers for cellular drug and gene delivery. One main synthesis route—template-directed synthesis—has been performed to prepare spherical LDH structure, involving either soft templates or hard templates. The soft templates, for example spherical vesicle formed by sodium dodecanesulfonate (SDS), were used to synthesize micrometer-sized rosette-like solid LDH spherical aggregates with SDS anions intercalated already in the LDH galleries, by urea hydrolysis under high-temperature hydrothermal treatment of 150 °C [16]. Coral-like MgAl-LDH solid microsphere was also synthesized using ethylene glycol/methanol/dodecyl sulfate nonaqueous polar solvent/surfactant system [13]. The hard template method, using hard cores such as polystyrene (PS) beads as templates, involves formation of the LDH hollow sphere with nanoshell face-on orientation. In such a method, positively charged colloidal LDH nanoplatelets were necessarily prepared, and then deposited or assembled as building block on the surface of PS latex [17], carbon [15], and silica [18] (Figure 3), giving the LDH shell walls. The necessity of preparing positively charged LDH was achieved either by synthesizing LDH nanoparticles using a protocol involving a fast coprecipitation followed by controlled hydrothermal treatment [11], or by obtaining LDH nanosheets via delamination of Al^3+^-based types of LDHs (such as CoAl-, ZnAl-LDH, and MgAl-LDH) in formamide [35,36,37,38]. Compared with the soft template approach, the hard template could have advantages in facile preparation of size-controllable hollow spheres due to the rich functional groups on the surface. 

Furthermore, note that with the aid of the template of 3-D close-packed PS array, a three-dimension open macroporous (3DOM) LDH framework was prepared during a process of coprecipitation [39,40].

### 2.3. 1-D LDHs Nanostructures

**Figure 4:**
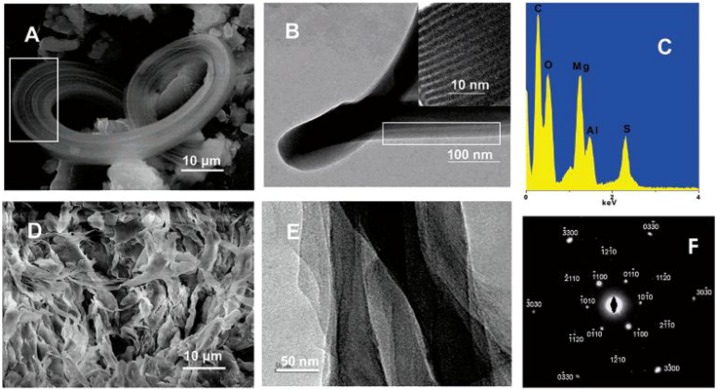
(**A**) SEM image of a belt-like LDH structure with the parallel linear pattern highlighted in the white rectangle. (**B**) TEM image of one belt reveals the lamellar structure; a HRTEM image of this structure is shown in the inset, and (**C**) spectrum of the chemical compositions analyzed by energy dispersive X-ray (EDX). The belts were easily exfoliated to give structures shown by the SEM image in (**D**) and TEM image in (**E**). (**F**) The corresponding selective area electron diffraction pattern. Reprinted with permission from [19], © American Chemical Society, 2005.

**Figure 5:**
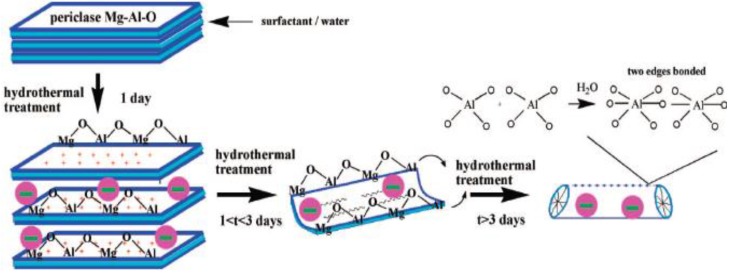
Possible mechanism for the folding of layers of LDH to give fibrous hydrotalcites. Reprinted with permission from [20], © American Chemical Society, 2008.

LDHs are well-known to show preferential growth perpendicular to the stacking direction, and therefore it is still a challenge to engineer microsized LDH platelet into 1-D structures. This type of rigid platelet is not similar to a graphene sheet which is able to be rolled into a tube. In few previous studies [41,42,43], 1-D LDH structures were obtained through the control of nucleation and growth of LDH crystal, such as nanobelt and fibrous structure. Zhang *et al.* synthesized MgAl-LDHs with a high aspect ratio via calcination and rehydrolysis under hydrothermal conditions [41]. Zhao *et al.* prepared MgAl-LDH nanowires and nanorods via hydrothermal treatment at a high temperature and ZnCoFe-LDH nanowires with controllable morphology in a water-in-oil microemulsion [42,43]. Interestingly, O’Hare’ group obtained MgAl-LDH belt structure in a water-in-oil reverse microemulsion system (Figure 4) [19]. The reverse system involved was considered to offer two important water pools in the confined nucleation and growth: small spheroidal microemulsion spontaneously formed by SDS act as microreactors for the nucleation and crystallization of LDHs, and large branching reservoirs contributing to confine LDH growth that is distinctly different from the traditional coprecipitation with a preferred orientation along the *c* axis [19]. Recently, Lima *et al.* fabricated fibular LDH via folding of LDH layers by the aid of SDS surfactant [20]. The possible mechanism is that mixed oxides ((Mg, Al)-O) recover the layered structure typical of hydrotalcites in the presence of SDS solutions, and then the most external layers in a particle of LDH fold to give rise to a mesostructured material by the formation of small amounts of Al-OH-Al groups (Figure 5). The local disturbance in the layered structure was thereby proposed as an evolution from platelet-like to fibrous pipe. Comparison of all the 1-D LDH structures in the above studies shows the dependence of the formation on relatively restricted conditions necessary to form vesicles, micelles, or reverse emulsions, since the weak liquid confinements are easily deformed and coalesced during synthesis.

### 2.4. 2-D LDH Films

LDH films have increasingly attracted attention due to advantages in the context of immobilization of LDH structures. One advantage of fabrication of well-oriented or self-supporting LDH films is to avoid spontaneous aggregation of LDH powders. Previous studies have shown the fabrications of LDH films with microsized LDH platelets oriented perpendicularly or horizontally to the underlying metal or surface-modified substrates [21,22,23,24,25,26,44,45,46,47,48,49,50,51,52,53,54,55,56,57,58,59,60,61,62]. With regards to orientation, 2-D LDH films can be classified into two main types: perpendicular and parallel oriented. 

#### 2.4.1. Parallel-Oriented LDH Films 

Parallel-oriented LDH films usually consist of face-on oriented building blocks of platelet-like LDHs. The face-on orientation of the building blocks of LDH platelets are achieved through various methods, such as solvent evaporation [44,45,46,47,48,49,50], electrochemical deposition [51,52,53], spin-coating technique [54], Langmuir-Blodgett (LB) technique [55], Layer-by-layer (LbL) technique [35,36,37,38], and *in-situ* growth [27]. 

**Figure 6:**
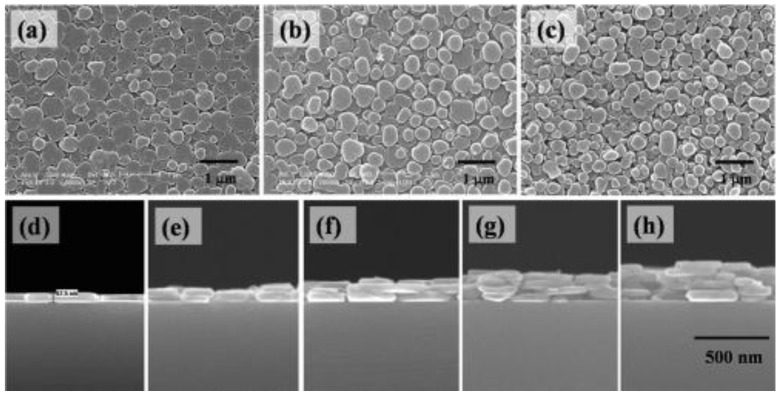
SEM images of the monolayer of LbL LDH nanocrystals: 1 layer (L) (**a**); 3 L (**b**); and 5 L MgAl-LDH/Si (**c**); Cross-sectional views of 1 L (**d**); 2 L (**e**); 3 L (**f**); 4 L (**g**) and 5 L (**h**) samples. Reprinted with permission from [49], © American Chemical Society, 2007.

Solvent evaporation and colloid deposition technique are the direct ways to prepare LDH films. LDH platelets or exfoliated LDH nanosheets are evenly dispersed in solvent (such as methanol, ethanol, and water) and assembled with the solvent evaporation. Iyi *et al.* prepared water-swellable MgAl-LDH film by solvent evaporation on polyethylene (PE) substrate and obtained self-supported film of thickness in the range from 10 to 25 µm [44]. Wang *et al*. fabricated transparent ZnAl- and NiAl-LDH films by solvent evaporation of LDH powder obtained by the SNAS method [45]. Using colloid deposition, Itaya *et al.* prepared film of 100 nm thickness on a SnO_2_ electrode [46]. Similarly, Lee *et al.* prepared the monolayer [47,48], the multilayer [49] (Figure 6), and transparent hybrid layer [50] on substrates—all were assembled from LDH nanoplatelets. Electrochemical deposition technique is also used to prepare LDH films due to the positive charge of LDH colloid. By the aid of this method, ZnAl-LDH, CoAl-LDH, and NiAl-LDH films were prepared on different electrode surfaces [51,52,53]. Spin coating is another effective approach for fabrication of inorganic films with controlled structure and crystal orientation. Zhang *et al*. reported that MgAl-LDH films was readily prepared by spin coating nanodispersed MgAl-LDH solution on a magnesium-containing alloy substrate [54]. In addition, LB technique was utilized to fabricate MgAl-LDH film using an anionic Ru(II) cyanide polypyridyl complex as template [55]. 

**Figure 7:**
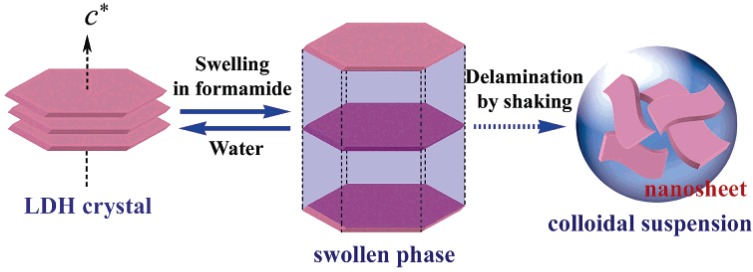
Schematic illustration of the possible delamination mechanism for LDHs in formamide, which were used as positively charged building block to prepare multilayer film of (LDH/PSS)n assembled on a quartz glass substrate. Reprinted with permission from [58], © American Chemical Society, 2006.

Compared with the above methods, LbL assembly is a commonly used approach to prepare LDH films. This approach is based on assembly of alternating deposition of oppositely charged building blocks either mainly via electrostatic interaction [35], to which Sasaki and co-workers have made a pioneer contribution, or via hydrogen bonding [38]. Through assembly of positively charged LDH building blocks and negatively charged polyelectrolytes, a serials of LDH nanosheet/polyelectrolyte heterogeneous ultrathin films were successfully prepared [35,36,37], such as CoAl-LDH/poly(sodium 4-styrenesulfonate) [35,37] (Figure 7) and MgAl-LDH/poly(p-phenylene) anionic derivate ultrathin films [36]. Typically, the LDH nanosheets utilized were obtained via exploitation of Al^3+^-based type (such as CoAl- and MgAl-LDH) in formamide, as mentioned in Section 2.2. 

*In situ* growth is also an effective method to fabricate parallel-oriented LDH films whose crystal is directly formed on the surface of the substrate. One feature of the approach lies in the strong adhesion between the LDHs and the substrates. We have recently shown a parallel-oriented LDH structure with LDH *c*-axis perpendicular to the glass substrate upon surface modification of poly(vinyl alcohol) (PVA) [27]. The X-Ray diffraction (XRD) pattern of the LDH film showed the absence of any non-basal reflections *(h, k* ≠ 0) at high angle, instead of basal (00*l*) reflections with a series of LDH peaks appearing as narrow, symmetric, strong lines at low angles. The hydrogen bonding between PVA and the hydroxyl groups of LDH crystallites orientation was proposed for the formation of the orientation. 

#### 2.4.2. Perpendicular-Oriented LDH Films 

**Figure 8:**
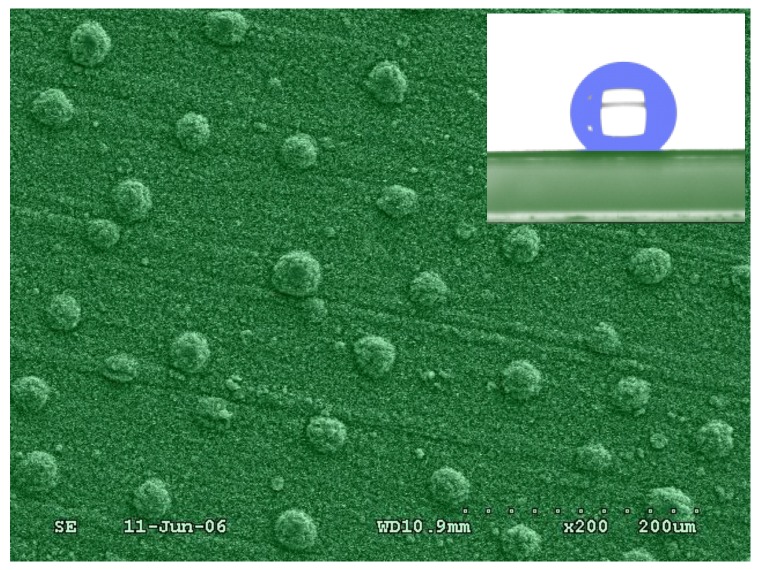
SEM of the ZnAl-LDH-laurate hybrid film showing the hemispherical protrusions. ZnAl-LDH-La/Al. The inset shows the image of contact angle of a water droplet on the surface of the ZnAl-LDH-laurate hybrid film.

Perpendicular-oriented LDH films are usually composed of edge-on oriented LDH platelets. Such films were typically prepared using *in situ* growth based on a hydrothermal synthesis, which is mentioned in Section 2.4.1 to fabricate parallel oriented LDH films. Lei *et al*. reported the edge-on oriented MgAl-LDH thin films with microscale thickness on the sulfonated PS flat substrates by urea hydrolysis reaction at 70–80 °C over more than eight days [56]. Further investigation of growth kinetics of LDH microcrystals supported the possibility of templating the formation of the edge-on orientated MgAl-LDH film [57]. Using the approach, Gao *et al*. reported the preparation of a ZnAl-LDH film on single Al substrate [22]. As an alternative, Chen *et al.* presented the preparation of NiAl-LDH films by using single porous anodic alumina/aluminum (PAO/Al) foil as both substrate and sole source of aluminum [21]. This opened up the possibility of growing large-scale uniform LDH films directly from substrates. Zhang *et al.* further demonstrated the possibilities of micro/nanostructured ZnAl-LDH film precursors, with (Figure 8) or without the necessity of the pre-treatment of anodization to Al substrate [25,26]. More interestingly, using two substrates (Al and Zn or Cu foils) as trivalent and bivalent resources, respectively, Huang’s group fabricated ZnAl- and CuAl-LDH films on the Al substrates [24]. Recently, Zhao *et al.* reported the fabrication of hierarchical ZnAl-, NiAl-, and MgAl-LDH films on intriguing man-made supports (article, cloth and sponge) through a combined procedure of sol-gel process and *in situ* growth method [58]. All the involved LDH structures observed by scanning electron microscope (SEM) in the above studies showed that the building blocks, typically microsized LDH platelets, were observed perpendicular to the underlying surfaces of the substrates. The XRD patterns involved, however, show the different results of the presence or absence of basal and higher order (00*l*) reflections at low angle. This could be attributed to the difference in orientations between vertically oriented and titled LDH platelets, which could deserve further research in the field of inorganic materials.

On the basis of the above-mentioned LDH micro-/nanostructures, an overview of the morphologies in terms of dimension is given in Table 1.

**Table 1:** Overview of the LDH micro-/nanostructures with different dimensions and synthesis.

Dimension	Morphology	Method	References
0-D	powder	SNAS, coprecipitation, hydrothermal	28-34
	sphere	LbL, coprecipitation	15-18
1-D	high aspect ratio	hydrothermal	41
	nanowire	calcination and rehydrolysis, hydrothermal	42, 43
	nanobelt	coprecipitation	19
	nanotube	coprecipitation	20
2-D	parallel-oriented	solvent evaporation, electrochemical deposition spin-coating, LB, LbL, *in-situ* growth	44-59
	perpendicular-oriented	*in-situ* growth	21-26, 60-62
3-D	3DOM	coprecipitation	39-40

## 3. Application of LDHs

The nanostructures involved often combine fascinating shapes with remarkable properties. With the introduction of new techniques and novel concepts for preparation of LDH structures, together with the requirements of practical applications, various studies focus on the properties and applications of nanostructured LDHs.

### 3.1. Catalysts

LDH powder, LDH film, and activated rehydrated LDH (RLDH) structures are well-known as effective solid catalysts. Zhang *et al.* used the Cu^2+^-based LDH powder as a catalyst for wet oxidation [59], and reported that the array of active Cu^2+^ centers on the surface of CuZnAl-CLDH is ordered net shaped, which is influenced by the effect of ordered cross trap. The advantages of *novel* LDH films are able to overcome the problems of use of powdery catalysts on an industrial scale, such as high pressure drop and difficult catalyst separation. Lv *et al.* reported that the activated MgAl-LDH film was promising to be used as precursor to synthesize monolithic catalyst for the aldol condensation of acetone and other base catalyzed reactions [60]. For the interesting 3DOM LDH structure, Géraud *et al.* demonstrated that the activated MgAl-LDH, with decatungstate anion intercalated exhibited the enhanced photocatalytic activity for the photodegradation of 2, 6-dimethylphenol with control of the corresponding coprecipitated LDH material [40], due to the high specific surface of the novel 3DOM structure.

### 3.2. Water Treatment

Waste water often contains oxyanions such as F^–^, Cl^–^, Br^–^, PO_4_^3–^, which are harmful to both humans and wildlife. Enhanced ability to remove oxyanions is of importance in environment protection. Structured LDHs are one new type of promising material due to their ability to capture organic and inorganic anions which can be used in water treatment. Calcined LDH powder is also an important type of material used in water treatment, since LDH can afford mixed metal oxides and has a special property called memory effect. The main advantages of LDHs over the conventional anionic exchange resins include their higher anion exchange capacity for certain oxyanions and their good thermal stability. Furthermore, LDHs can be fully regenerated in a short time for reuse. Lv *et al.* used MgAl-NO_3_-LDH powder [61] and calcined powder [62] to remove F^–^, Cl^–^, Br^–^, and I^–^, and concluded that the rate constant for exchange of nitrate anions by halide decreases in the order of F^–^ > Cl^–^ > Br^–^ > I^–^, following pseudo-second order kinetic models. As described above in Section 2.4, the edge-on LDH films prepared on man-made supports exhibited the enhanced performances of regeneration for water treatment and membrane separation in comparison with the corresponding powdery LDH [58]. The novel and well-dispersed nanocrystals of LDH on the paper substrate without aggregation was considered to provide large specific surface area as well as good accessibility, underlying the superior adsorption performances of the film sample [58].

### 3.3. Additives in Concrete and Flame Retardants

Powdered LDH are one types of additives in concrete. Basically, CaAl-LDH has a general formula very similar to the AFm phase occurring in hydrated cement, being composed of positively charged layers [Ca_2_Al(OH)_6_]^+^, and negatively charged interlayers [X^−^·*n*H_2_O]^−^. AFm phase is generally believed to act as a crystal seed accelerating cement hydration, CaAl-LDHs have been, therefore, proposed as potential concrete hardening accelerators. Raki *et al.* synthesized CaAl-LDHs intercalated with nitrobenzoic acid, naphthalene-2, 6-disulfonic acid, and naphthalene-2 sulfonic acid, which were promising for future applications in cement and concrete science [63]. We recently reported that the specimens containing pure CaAl-LDHs, free of contamination by CaCO_3_, exhibited a greatly enhanced performance in respect to early compressive strength and early flexural strength; and the values increased by 61% and 71%, respectively, compared to the pristine concrete specimen [34]. 

Powdery LDH is also commercially promising as an additive in flame retardants. Many flame retardants are considered harmful, having been linked to liver, thyroid, reproductive/developmental, and neurological effects. Currently, halogen-free alternatives are one active research area. LDHs and cationic clays (e.g., montmorillonite) have been widely investigated as additives in this context. Compared to other flame retardants, LDH is a new type of material due to high smoke suppression, nontoxicity or low toxicity. LDH is currently explored as a second generation flame retardant with enhanced properties by either modification of the layers or intercalation of different anions. Lin *et al.* found that a borate-pillared MgAl-LDH with ethylene vinyl acetate (EVA) as the polymer component was a promising flame retardant [64]. By tuning the composition of the layers, the flame retardancy of LDHs was steered. For example, ternary ZnMgAl-LDH showed better flame retarding properties than binary MgAl-CO_3_^2–^-LDHs [65]. 

### 3.4. LDHs in Biology and Medicine

Powdered LDHs have demonstrated to be one type of important and green carrier or host for genes and drugs due to the excellent biocompatibility and nontoxicity or low toxicity. MgAl-LDH is used as an important component of drugs, or as nanocarriers for delivery of drug and genes into cells [5,8]. Wei *et al.* demonstrated that LDH was able to be used as an effective nanocarrier by greatly enhancing the thermal- and photo-stabilities for L-Dopa [66] and L-Tyrosine [67], which are both unstable agents in storage or transport. Comprehensive investigations have been performed in Choy’s group on the toxicity of LDH nanoparticles *in vitro* and *in vivo* in practical biological applications [5]. Interestingly, Lu’s group recently reported an efficient LDH-based delivery for siRNA to mammalian cells *in vitro*. A pronounced down-regulation of protein expression upon LDH mediated siRNA transfection of HEK293T cells was observed [68]. 

## 4. Conclusions 

In this feature article we have reviewed the morphology, preparation, and application of LDH structures. Basically, micro-/nanosized LDH powder is prepared through hydrothermal synthesis, conventional coprecipitation or scalable SNAS. The edge-on oriented LDH spheres (perpendicular to the tangent plane at the spherical surface) and films are fabricated via *in situ* growth, which is actually performed on the basis of hydrothermal synthesis, whereas the preparation of the face-on oriented LDH spheres and films is achieved by physical deposition methods (mainly consisting of LbL assembly, sol-gel spin-coating and the solvent evaporation technique) and hydrogen-bonding-based *in situ* growth. 1-D structured LDHs can be synthesized via control of LDH nucleation and growth in confined microsystems. In the point of view of applications, LDH powders and films are widely explored as catalysts and adsorbed materials, upon calcination and rehydration, the resulting activated structures exhibit enhanced performances. Powdery LDHs are typically used as addictives in flame retardants and concrete, and as nanocarriers for drugs, gene molecules, biomedical products, and functional molecules. A few big challenges, however, still remain, involving dedication to the problem of well-dispersed LDH nanoplatelets as additives in flame retardants and also to realize non-toxic, biocompatible and biodegradable LDH carries into practical applications for drug and gene delivery. Some perspectives could focus on preparing novel LDH micro-/nanostructures and developing novel applications in many fields such as separation, catalyst, drug and gene delivery, and electrode modifier energy harvesting devices, and also on the relationship between the novel structures and the enhanced properties of LDH micro-/nanostructures, as well as further understanding the specific structures prepared from confined nucleation and growth. These aspects would advance our knowledge of control LDH nanostructures, resultant properties and applications. 
